# Base excision DNA repair and cancer

**DOI:** 10.18632/oncotarget.2705

**Published:** 2014-12-09

**Authors:** Gianluca Tell, Bruce Demple

**Affiliations:** Department of Medical and Biological Sciences, University of Udine, Udine 33100, Italy

Transformed cells can develop drug resistance via repair mechanisms that counteract the DNA damage from chemotherapy or radiation therapy. Disruption of DNA repair pathways can cause mis-repair that is cytotoxic [1]. Specific DNA repair inhibitors might thus be combined with DNA-damaging agents for improved therapy. In addition, some cancer cells have a reduced repertoire of DNA damage responses, which provides other therapeutic openings. Recent studies show that many DNA repair proteins are associated with those involved in RNA metabolism and transcriptional regulation, including within the nucleolus [2].

The base excision DNA repair (BER) pathway handles simple alkylation and oxidative lesions arising from both endogenous and exogenous sources, including cancer therapy agents. The core BER enzyme Ape1 also contributes to the regulation of oxidative stress responses and has other non-repair activities, such as regulating the expression of chemoresistance genes (i.e. MDR1) [3]. Ape1 is thus an emerging target for combination therapy of different cancers.

Ape1 can function as a “redox factor” [1] that stimulates DNA binding by transcription factors involved in cancer promotion and progression, such as NF-ĸB, Egr-1, Hif-1α, Nrf1 [3], thus influencing inflammatory and metastatic processes. A third poorly characterized Ape1 function is its transcriptional activity on genes such as SIRT1 and those encoding some mitochondrial proteins (*Tfam, Cox6c,* and *Tomm22*) [2]. Moreover, Ape1 regulates the expression of tumor-progression and therapy-resistance genes through transcriptional effects (on the VEGF and MDR1 genes, for example) and post-transcriptional mechanisms through direct mRNA binding by Ape1 (e.g. c-Myc) [3]. These observations prompt a new model that links DNA damage responses and the modulation of target genes, which may provide chemoresistance during tumor development.

**Figure 1:**
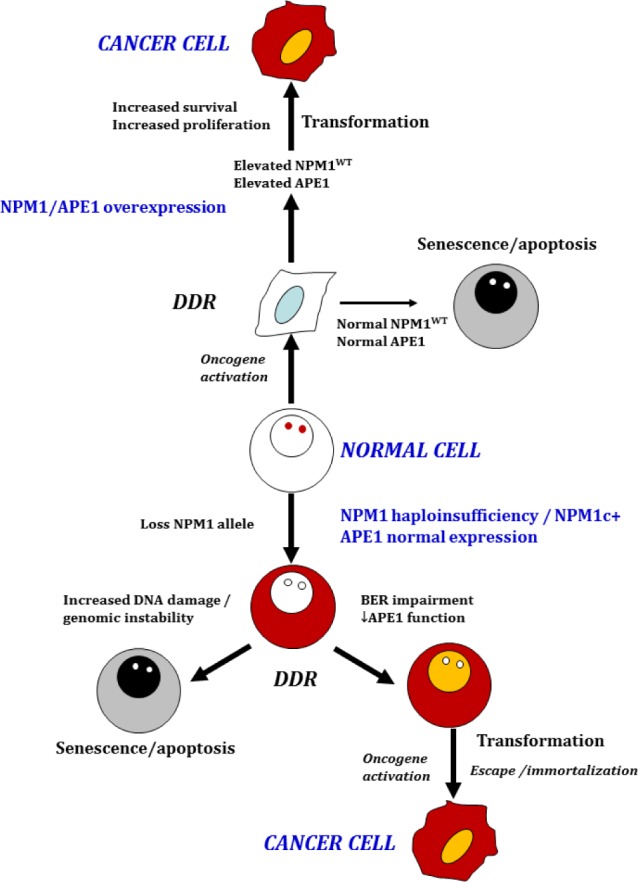
The relative contribution of NPM1 and Ape1 expression to cellular transformation: a new model for the role of Ape1 in tumorigenesis

Cancer-associated Ape1 variants are often altered in the protein's DNA repair domain, with some exhibiting nuclease defects *in vitro* [4]. Up-regulation of Ape1 correlates with the onset of chemoresistance in ovarian, hepatic and neurologic tumors, while inhibition of the protein with small compounds, or the downregulation of its expression, sensitizes cells to DNA-damaging chemotherapeutic drugs and ionizing radiation [3]. Which Ape1 activity is involved in cancer development or chemoresistance remains unknown. We discovered a function of Ape1 in rRNA metabolism involving direct rRNA binding and interaction with NPM1, which is required for retaining Ape1 in the nucleolus. A role in rRNA metabolism may explain the altered Ape1 expression observed in different tumors [3]. Although knowledge of Ape1's possible function in the nucleolus is incomplete, the protein retained there may provide an immediate source of additional enzyme for BER in cells subjected to genotoxic stress. The interaction with NPM1 also stimulates Ape1 DNA repair activity; accordingly, NPM1^−/−^ cells show impaired BER activity [5]. The NPMc+ mutation (frequently found in blasts from AML patients) relocalizes the protein to the cytoplasm taking Ape1 with it [7]. NPM1c+ tumors have a good prognosis for chemotherapy [3], perhaps related to nuclear deficiency of Ape1. Conversely, in solid tumors such as hepatic and ovarian cancers, Ape1 and NPM1 up-regulation is linked to increased chemoresistance [3]. Even without a genotoxic challenge, cell lines expressing an Ape1 variant that does not interact with NPM1 display reduced proliferation [5]. Thus, the Ape1-NPM1 association highlights the role of Ape1 dysregulation in cancer biology (Fig. 1). Reduced NPM1 levels may lead to genomic instability through impairment of BER and increased DNA damage. As a consequence, the DNA damage response blocks cellular proliferation. A few cells may escape the blockage and establish an immortalized clone susceptible to oncogenic transformation. Alternatively, the presence of high NPM1 and Ape1 levels may limit DNA damage and the DNA damage response, thus supporting cell survival and generating a permissive condition for transformation. These studies further suggest that other alterations of the Ape1 interactome may lead to the impairment of BER as observed for the NPMc+ mutation. We identified novel Ape1 acetylation sites dysregulated in TNBC, responsible for stimulating the endonuclease activity of the protein and its binding to NPM1 and RNA molecules [5]. Therefore, the modulation of Ape1 modifications and interactions affects BER activity and is linked to tumorigenesis.

In the last decade, several laboratories identified inhibitors of the Ref-1 redox activity (e.g., E3330, RN81 and resveratrol), or inhibitors of the Ape1 DNA repair activity (i.e. lucanthone, CRT0044876, myricetin, etc.) [3]. However, these compounds have limited specificity: the activities are not unique to transformed cells. A recently identified role of Ape1 in telomere maintenance [7] might provide other interactions that could be usefully disrupted in cancer cells.

Because multiple cellular functions can be affected by inhibiting Ape1, targeting its interactions with other proteins such as NPM1 may represent a powerful strategy for developing more specific anticancer drugs. Therefore, current research must focus on understanding all the roles of Ape1 in cancer resistance, including its role in the nucleolus, and the fine-tuning mechanisms responsible for regulation of these activities.
